# Cytotoxic and antimicrobial activities of endophytic fungi isolated from *Bacopa monnieri* (L.) Pennell (Scrophulariaceae)

**DOI:** 10.1186/1472-6882-14-52

**Published:** 2014-02-11

**Authors:** Meenu Katoch, Gurpreet Singh, Sadhna Sharma, Nidhi Gupta, Payare Lal Sangwan, Ajit Kumar Saxena

**Affiliations:** 1 Microbial Biotechnology Department, Indian Institute of Integrative Medicine, Jammu, India; 2 Cancer Pharmacology Department, Indian Institute of Integrative Medicine, Jammu, India; 3 Natural Product Chemistry, Indian Institute of Integrative Medicine, Jammu 180001, India

**Keywords:** *Bacopa monnieri*, Endophytic fungi, MTT assay, Antimicrobial activity, Cytotoxicity, HCT-116

## Abstract

**Background:**

Endophytes, which reside in plant tissues, have the potential to produce novel metabolites with immense benefits for health industry. Cytotoxic and antimicrobial activities of endophytic fungi isolated from *Bacopa monnieri* (**L.**) Pennell were investigated.

**Methods:**

Endophytic fungi were isolated from the *Bacopa monnieri*. Extracts from liquid cultures were tested for cytotoxicity against a number of cancer cell lines using the MTT assay. Antimicrobial activity was determined using the micro dilution method.

**Results:**

22% of the examined extracts showed potent (IC_50_ of <20 μg/ml) cytotoxic activity against HCT-116 cell line. 5.5%, 11%, 11% of the extracts were found to be cytotoxic for MCF-7, PC-3, and A-549 cell lines respectively. 33% extracts displayed antimicrobial activity against at least one test organism with MIC value 10–100 μg/ml. The isolate B9_Pink showed the most potent cytotoxic activity for all the cell lines examined and maximum antimicrobial activity against the four pathogens examined which was followed by B19.

**Conclusions:**

Results indicated the potential for production of bioactive agents from endophytes of *Bacopa monnieri*.

## Background

The antibiotic resistance of bacterial pathogens has become a serious health concern. Therefore, there is an imminent need to explore the potential of novel, naturally occurring antimicrobial metabolites [1].

Endophytic fungi asymptomatically reside in the internal tissues of plants [2]. Their biological diversity, especially in temperate and tropical rainforests, is enormous [3]. The fungi are found in nearly 300,000 land plant species, with each plant hosting one or more of these fungi [4]. Endophytic fungi have been isolated from many different plants including trees (pine and yew), fodders (alfa-alfa, sorghum, and clover), vegetables (carrot, radish, tomatoes, sweet potatoes, lettuce, and soyabean), fruits (banana, pineapple, and citrus), cereal grain (maize, rice, and wheat), and other crops (sugarcane, marigold, and coffee) [5]. Since endophytes exhibit a rich diversity, biological novelty is to be expected [6].

The endophytic fungi associated with medicinal plants not only produce antibacterial molecules but also many other pharmacologically active substances with a potential to act as antitumor agents (*Pestalotiopsis microspora*, taxol), antifungal agents (*Cryptosporiopsis criptocandina*, quercine) [4,5,7]. Moreover, endophytes are also known to produce factors for plant growth, toxins and enzymes. Some endophytes are also being used as biological controllers of many diseases and plaques [7,8]. An increasing number of compounds have been isolated from different endophytic fungi for example, fumitremorgins B has been isolated from *Phomopsis sp*. and periconicins A and B have been isolated from *Periconia sp*[9,10]. The potential for isolation of novel, natural, and potent metabolites from unexplored endophytes should encourage research in this field.

*Bacopa monnieri* (**L.**) Pennell (Scrophulariaceae), commonly known as “Brami” or Indian water hyssop is commonly found in Asia, Australia, and America. Since time immemorial, it has been used in ayurvedic formulations for treating gastrointestinal and neurologic disorders [11]. Recent studies have proved that its active constituent bacosides enhances the efficiency of nerve impulse transmission leading to improve memory related functions. It also has hepatoprotective, antidepressant, and antioxidant properties [12-14]. Moreover, there are no previous reports on the isolation and cultivation of endophytes from *Bacopa monnieri*. Therefore, the current study was undertaken to isolate endophytic fungi from *Bacopa monnieri*, and screen them for the presence of any cytotoxic and antibacterial activity.

## Methods

### Plant material as source of endophytic fungi

Plant material was collected randomly from fully matured *Bacopa monnieri* between March – April, 2011 from the farm of IIIM, Chatha, at an altitude of about 32.73°N 74.87°E in Jammu and Kashmir State, in India. This accession is collected from Miran Sahib, Jammu in 1970. The identification of species was done via leaf and flower morphology by taxonomist of the Institute (IIIM) (Dr B. M. Sharma). An identified specimen was kept in the IIIM Janaki Ammal Herbarium in India with accession no. 18554. The accession is also maintained as genetic resource in the Chatha farm of IIIM. After plant selection, disease free leaves and branches of the plant were excised with a sterile scalpel and were sealed with parafilm to preclude drying out during transport.

### Isolation of endophytes

Isolation of endophytic fungi from *Bacopa monnieri* was carried out using the protocol described by Strobel and Daisy [4] with slight modifications. Fresh plant material (Branches and leaves) was collected. The leaves and small branches were washed under running tap water for 10 minutes and sterilized in series with 70% ethanol for 1 min, 1.0% sodium hypochlorite (NaOCl) (v/v) for 1 min and further cleaned by passing through two sets of sterile distilled water. After surface sterilization, leaves and branches were cut into small pieces, 1 cm long each, The sterile samples were placed on a plate containing water agar and potato dextrose agar (PDA) media with 250 μg/mL streptomycin to suppress bacterial contamination. The parafilm wrapped petri dishes were incubated at 25 ± 2°C till the fungal mycelia started growing on the samples. The endophytic fungi were transferred to a new potato dextrose agar slant. Endophytic fungi isolated from the leaves of *Bacopa monnieri* was codified as B1-B6, B8-B11, B13-B16, B18-B19, B22-B24, B9_Pink, B8_ORG, BX. Endophytes were stored at 4°C and also endophyte colonized on sterile barley seeds were air dried to be subsequently stored at −70°C for later studies. All the isolated endophytic fungi were deposited in Microbial Repository of IIIM.

### Fermentation and extraction

The endophytic fungi isolates (nine) were cultured in potato dextrose liquid medium in 1000 mL Erlenmeyer flask for a period of 10 days at 25 ± 2°C at 180 rpm on an incubatory shaker (New brunswicks, USA). Ten blocks containing 10-day old fungal mycelium were used as inoculums. After ten days, crude fermentation broth was blended thoroughly in a cell disintegrator with 20% methanol. Cell homogenate was extracted four times with equal volume of DCM (HPLC grade). Solvent was stripped off in a rotary evaporator leaving behind a residue designated as organic residue (O). The retentate was filtered and supernatant was lyophilized and designated as water extract (A). The extracts were dissolved in DMSO to a final concentration of 10 mg/ml for anticancer and antimicrobial activity screening.

### Cytotoxic activity

This assay is a quantitative colorimetric method for determination of cell survival and proliferation [15]. For *in-vitro* cytotoxic activity, colorectal carcinoma HCT-116, lung A-549, Breast MCF-7, prostate PC-3 cancer cell lines were procured from National Centre for Cell Sciences (NCCS), Pune, India. Cells were grown in RPMI-1640 medium containing 10% FCS, 100 U penicillin/100 μg per mL streptomycin in CO_2_ incubator (Thermo-con Electron Corporation, USA) at 37°C with 98% humidity and 5% CO_2_ gas environment. The cells were plated in a 96-well plate at a density of 2.0 × 10^4^ in 200 μL of medium per well. Cultures were incubated with different concentrations of fungal extracts (10–100 μg per mL) for 48 h. The medium was replaced with fresh medium containing 100 μg per mL of 3-(4, 5-dimethylthiazol-2-yl)-2, 5-diphenyltetrazolium bromide (MTT) and plates were incubated for 3 h. The supernatant was aspirated and MTT-formazan crystals were dissolved in 200 μL DMSO and the OD of the resulting solution was measured at λ_540nm_ (reference wavelength, λ_620nm_) on an ELISA reader (Thermo Labs, USA). Cell growth was calculated by comparing the absorbance of treated versus untreated cells. Cells treated with equivalent concentration of DMSO were used as negative control. Clinical drugs like 5-Fu, paclitaxel, adriamycin were included as positive controls. IC_50_ value was calculated by Curvfit software.

### Antimicrobial activity

Antimicrobial activity was determined using the micro dilution method [16]. Lyophilized six test bacteria and a yeast were purchased from Microbial Type Culture Collection (MTCC). Cultures of *Bacillus subtilis* (MTCC No. 121), *Pseudomonas aeruginosa* (MTCC No. 424), *Salmonella typhimurium* (MTCC No. 98), *Escherichia coli* (MTCC No. 118), *Klebsiella pneumonia* (MTCC No. 109), *Staphylococcus aureus* (MTCC No. 737) were grown on Nutrient Agar media and used for measuring the antibacterial activity of isolated endophytes. *Candida albicans* (MTCC No. 183) was grown on Yeast extract Peptone Dextrose Agar (YEPD).

Each bacterial strain was inoculated into nutrient broth (HiMedia Biosciences) and incubated overnight at 37°C with shaking. The suspension was adjusted to 0.5 McFarland standard turbidity (equivalent to 1.5 × 10^8^ colony forming units (CFU/ mL) [17] and finally diluted to give approximately 10^4^ CFU/mL for all organisms. Different dilutions (10–100 μg/mL) were prepared from the stock solutions of fungal extracts (10 mg/ml) and antibiotics (1 mg/ml). 900 μL of each concentration was mixed with 100 μL of sterile nutrient broth containing 10^4^ CFU bacteria, (obtained from a McFarland turbidity standard no. 0.5). Appropriate negative (DMSO-0.5%) and blank controls (virgin media) were used. Antibiotics like streptomycin, amphotericin B were included as positive controls. They were dissolved in sterile distilled water to a final concentration of 1 mg/mL. Tubes were incubated overnight at 37°C. The highest dilution at which 99.9% of the bacteria inoculum was killed was considered as the MBC and the lowest inhibitory dilution at which there was no visible growth was considered as MIC. The assays were replicated and the mean value of 3 experiments were recorded (n = 3) with SEM.

### Identification of potential endophytes

#### Morphological examination of endophytes

The fungi were identified based on the morphological characteristics. Colony features were based on observation on PDA under ambient day light conditions. Endophytes were identified on the basis of microscopic characteristics such as the structure of hyphae, conidia, and conidiophores. Conidiophore structure and morphology was ascertained after obtaining them from the edge of conidiogenous pustules or fascicles during maturation of conidia, which usually occurred after 4–7 days of incubation.

#### Internal transcribed spacer (ITS)-based identification

Endophytes were also identified by acquisition of the ITS 5.8S ribosomal gene sequence. The fungus was grown on PDA for 7 days. DNA was extracted following the protocol of Reader & Broda [18]. The ITS region of the fungus was amplified with the universal ITS primers ITS1 (5′-GGAAGTAAAAGTCGTAACAAGG-3′) and ITS2 (3′-TCCTCCGCTTATTGATATGC-5′) using PCR [19]. PCR was done as follows: 5 min at 95°C followed by 35 cycles of 94°C for 30s, 55°C for 1 min, 72°C for 1 min and 30s and a final extension for 10 min at 72°C. The 50-μL reaction mixture contained 1–10 ng of DNA, 1× PCR buffer (with 15 mM MgCl_2_), 200 mM of each dNTP, 10 pmol of each primer (Sigma, USA) and 1U *Taq* DNA polymerase (Bangalore Genei, India). The amplified product (10 μL) was resolved on 1% (w/v) agarose gel at 100 V. The amplified product (approx. 500 bp) was eluted using a Gel extraction Kit (Qiagen, USA) and 40–60 ng was used in a 10 μL sequencing reaction using Big Dye Terminator sequencing kit (v. 3.1, Applied Biosystems). The PCR forward/reserve primer (3.2 pmol) was used in cycle sequencing reaction. Samples were loaded on an automated sequencer (Applied Biosystems). The amplified products were sequenced. Resultant sequences (KF6839107, KF6839108, KF6839116, KF6839117, KF683919) were submitted to a gene bank and they were aligned with the sequences in the Gen Bank database via the BLASTn tool of NCBI [http://www.ncbi.nlm.nih.gov/Blast.cgi] [20]. Relevant sequences were downloaded and aligned using the MEGALIGN program (DNASTAR, Lasergene) and a phylogenetic tree and distance matrix was constructed according to Guindon and Gascuel [21].

## Results and discussion

A total of twenty six endophytes were isolated from *Bacopa monnieri*. Ten of them were identified on the basis of colony morphology and microscopic characteristic like the structure of hyphae, conidia, and conidiophores. Rest sixteen endophytes could not be identified on the basis of morphology. Randomly nine endophytes were chosen for the present study including three identified and six unidentified ones. Identified endophytes were B9_Pink, B16 (*Fusarium spp.*) and B4 (*Trichoderma sp*.) (Table 1). Unidentified endophytes were found to be unique one. Therefore it can be speculated that these unique endophytes might produce unique/potential bio-actives. Selected nine endophytes were fermented in potato dextrose broth for 10 days and processed to obtain organic and water extracts, which were then used for assessing the cytotoxic and antimicrobial activity.

**Table 1:** **Morphological and ITS-5.8S rRNA gene based identification of endophytic fungi isolated from *Bacopa monnieri***

**S No.**	**Endophytes**	**Source plant**	**Identified based on morphology**	**Identified with ITS based sequencing**	**Percent homology**
1	B1	*Bacopa monnieri*	-	-	-
2	B3		-	*Flavodon flavus*	99
3	B4		*Trichoderma sp.*	*Trichoderma aureoviride*	99
4	B9_pink		*Fusarium sp.*	*Fusarium sp.* 6241	99
5	B16		*Fusarium oxysporum*	*Fusarium oxysporum* isolate F1TK1	100
6	B19		-	*Fomitopsis cf. meliae*	100
7	B20		**-**	**-**	**-**
8	BX1		-	-	-
9	CK01		-	*Pleosporales sp.*	98

### Cytotoxic activity

Cytotoxicity of the extracts against HCT-116, MCF-7, PC-3, and A-549 cell lines is shown in Table 2. Extracts were found to be more effective against HCT-116 cells than the MCF-7, PC-3, and A-549 cell lines. Nearly one fifth (22%) of the extracts showed cytotoxic activity with IC_50_ of <20 μg/ml against HCT-116 cell lines, whereas only 5.5%, 11%, 11% of the extracts were found to be effective against MCF-7, PC-3, and A-549 cell lines respectively. These values were within the cut off point of the National Cancer Institute’s criteria for cytotoxicity (IC_50_ of <20 μg/ml) in the screening of crude plant extracts [22]. The isolate B9_Pink showed the most potent cytotoxic activity for all the cell lines examined, which was followed by B19. Thus the two isolates showed the potential to be used as anticancer drugs and needed to be further investigated.

**Table 2:** **Cytotoxic Activity (IC**_**50 **_**value in μg/ml) of the endophytic fungi isolated from *Bacopa monnieri***

**S No.**	**Endophytes**	**HCT-116**	**MCF-7**	**PC3**	**A549**
1	B1O	>100	0	>100	>100
2	B1A	>100	>100	>100	98.93
3	B3O	100	30	>100	100
4	B4O	**11**	>100	23	27
5	B4A	>100	>100	88.22	>100
6	B9_PinkO	5	7	**8**	**5**
7	B16O	22	>100	37	38
8	B16A	98.68	>100	>100	>100
9	B19O	**6**	>100	7	**6**
10	B19A	**15**	51	41	25
11	B20O	>100	0	>100	>100
12	B20A	>100	0	0	>100
13	BX1O	>100	>100	>100	>100
14	BX1A	>100	>100	>100	72.34
15	CK01O	**12**	>100	71	69
16	CK01A	>100	>100	>100	>100
17	Paclitaxel*	64	**18**	53	56

SD- ±0.2-1.4. IC_50_ value <20 μg/ml were given in boldface.

*Concentration of paclitaxel is 1 μM.

When tested against HCT-116 cell line, B9_PinkO (IC_50_ = 5 μg/ml) and B19O (IC_50_ = 6 μg/ml) extracts were found to be more toxic than chaetominine (IC_50_ = 11.3 μg/ml) isolated from the endophyte of *Adenophora axillifora* and less toxic than rubrofusarin B (IC_50_ = 4.5 μg/ml), isolated from the endophyte of *Cyndon dactylon* and cisplatin (Mayne Pharma) (IC_50_ = 0.6 μg/ml) [23,24].

Similarly, B9_PinkO (IC_50_ = 7 μg/ml) was found to be cytotoxic against MCF-7 cell line. Although its IC_50_ value is less than 10 μg/ml, but it was found to be less toxic than tamoxifen (Dynapharm), beauvericin (IC_50_ = 1.42 μg/ml) and bikaverin (IC_50_ = 0.161 μg/ml) isolated from an endophytic *Fusarium oxysporum*[25]. Carvalho et al. [26] also reported that nearly 80% of the endophytic extracts, which were isolated from plant *Stryphnodindron adstringens* (Mart.) Coville (Fabaceae), have IC_50_ of <20 μg/ml against MCF-7 cell line and only four extracts, out of these, had an IC_50_ of <10 μg/ml.

When tested against A-549 cell line, B9_PinkO (IC_50_ = 5 μg/ml) and B19O (IC_50_ = 6 μg/ml) extracts were found to be more toxic than the extract of *Hypocrea lixii* R18, an endophyte isolated from pigeon pea and cajanol, a molecule isolated from endophyte *Hypocrea lixii* R18 (with same IC_50_ = 20.5 μg/ml) [27]. In contrast, B9_PinkO and B19O extracts were found to be less toxic for A-549 cell-line than extracts of AM07 (IC_50_ = 2 μg/ml) and AM11 IC_50_ = (3.14 μg/ml), which are the endophytes from *Actinidia macrosperma*[28]. When tested against PC-3 cell line, B9_PinkO (IC_50_ = 7 μg/ml) and B19O (IC_50_ = 8 μg/ml) extracts were found to be more toxic than the extract of endophyte *Hypocrea lixii* R18 (IC_50_ = 29.8 μg/ml) isolated from pigeon pea [27]. Phongpaichit et al., [29] screened extracts from 41 endophytic fungi isolated from *Garcinia* plant and 33% of the screened extracts, showed cytotoxic activity (IC_50_ of <10 μg/ml) for Vero cell line. In contrast, Guimaraes et al. [30] screened extracts from 39 endophytic fungi isolated from *Viguiera arenaria* and *Tithonia diversifolia* and all of the extracts was found to be cytotoxic with IC_50_ of >20 μg/ml for JURKAT cell line.

### Antimicrobial activity

The anti-microbial activity of the extracts was tested against representative Gram positive and Gram negative bacteria, and yeast by tube dilution method. Results of antimicrobial activity are presented in Table 3. Only six extracts (30%) displayed antimicrobial activity against the tested organisms (MIC value 10–100 μg/ml). Approximately more than half of the active extracts (5) displayed antimicrobial activity against *S. typhimurium.* Aqueous extract of B19 was found to be microbicidal for *S. typhimurium*. Organic extracts of B9_Pink and CK01 isolates were found to be more potent than streptomycin against *S. typhimurium*. Organic extract of B9_Pink was also found to inhibit *E. coli, P. aeruginosa,* and *S. aureus* with an MIC/MBC of 10 μg/ml. Similarly, Organic extract of B19 inhibited the growth of all the tested pathogens (MIC/MBC 10–100 μg/ml). The Organic extract of B9_Pink and B19 were also found to be more potent than streptomycin against *E. coli*. In contrast to the present results, no extract of endophytic fungi isolated from *Ophiopogon japonicas* displayed inhibitory activity against *E. coli*[31]. Most of the other studies have shown that endophytes are a good source of antibacterial agents. Guimaraes et al. [30] screened extracts from 39 endophytic fungi isolated from *Viguiera arenaria* and *Tithonia diversifolia*, and found that 5.1% of the extracts were inhibitory against *S. aureus* and 25.6% extracts were inhibitory against *E. coli*. In the present study, antimicrobial activity of extracts in terms of MIC values was ranged between 10–100 μg/ml, whereas in another study, only two endophytic extracts showed MIC value less than 10 μg/ml against *Microsporum gypseum*[29].

**Table 3:** **MIC/MBC values (μg/mL) of the extracts prepared from endophytic fungi isolated from *Bacopa monnieri***

**S No.**	**Endophyte**	***E. coli***	***S. typhimurium***	***P. aeruginosa***	***S. aureus***	***K. pneumonia***	***C. albicans***
1	B1O	-	-	-	-	-	-
2	B1A	-	-	-	-	-	-
3	B3O	**10(MBC)**	-	-	-	-	-
4	B4O	-	-	**-**	**-**	**-**	**-**
5	B4A	-	-	-	-	-	-
6	B9_Pink	**10(MBC)**	**10**	**10(MBC)**	**10**	**-**	**-**
7	B16O	-	-	**-**	**-**	**-**	**-**
8	B16A	-	-	-	-	-	-
9	B19O	**10**	30	**10**	**10**	**30**	**100**
10	B19A	-	100	**-**	**-**	**-**	**-**
11	B20O	-	-	-	-	-	-
12	B20A	-	-	-	-	-	-
13	BX1O	-	-	-	**10**	-	-
14	BX1A	-	-	-	-	-	-
15	CK01O	**10**	**10**	**10**	**10**	-	-
16	CK01A	-	-	-	-	-	-
17	Streptomycin/Amphotericin	**10(MBC)**	**10**	**10(MBC)**	**10(MBC)**	**10(MBC)**	**0.625**

MIC/MBC value <20 μg/ml were given in boldface.

Endophytes have already been reported as being prolific producers of antimicrobial compounds. Many studies have indicated that *Fusarium spp.* are the most common species among endophytes from medicinal plants and a potent source of bioactive compounds. Antimicrobial compounds like the penta-ketide (CR377: 2-methylbutyraldehyde-substituted-α-pyrone), beauvericin, subglutinol A and B were isolated from *Fusrarium spp.,* endophytes of plants *Selaginella pallescens, Cinnamomum kanehirae, Tripterygium wilfordii* respectively. These compounds showed strong antimicrobial activity against *C. albicans,* and methicillin-resistant *S. aureus*[32-34]. Similarly altersetin from *Alternaria sp.,* phomoxanthone A and phomoxanthone B, and dicerandrols A-C from *Phomopsis sp.* endophytes showed significant antibacterial activities [35-37]. Similarly, antibacterial guanacasterpenes A-O were produced by an unidentified endophyte isolated from Costa Rican plant [38]. Guanacasterpene A showed antibacterial activity against meticillin-resistant *S. aureus* and vancomycin-resistant *Enterococcus faecium*[39], while guanacasterpene I showed pronounced activity against *S. aureus*[38].

Endophytes produce natural products for protecting the plant host against pests and pathogens [40]. In fact, the relationship between an endophyte and host plant is symbiotic. Natural selection is expected to favor those endophytic strains that produce defensive chemicals for their hosts. Furthermore, use of antimicrobial metabolites produced by the endophytes has many advantages such as, they are biodegradable, easy to be produced on a large scale, pose negligible threat to the ecology. Hence these should be exploited industrially.

**Figure 1:**
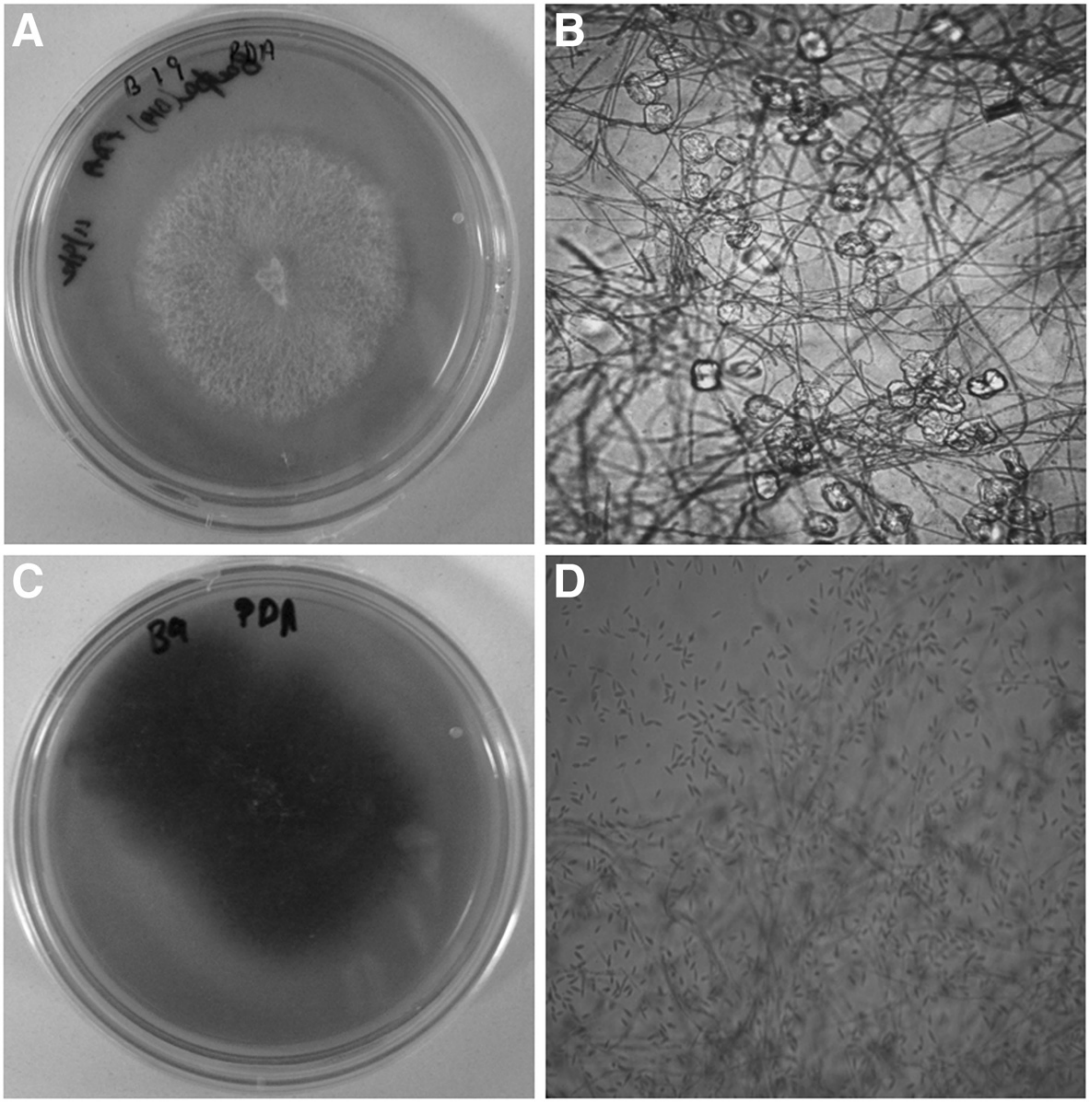
**Colony morphology and light microscopy of B9_Pink and B19 endophytic fungi associated with *Bacopa monnieri *** **A) B19 Colony, B) light microscopy of B19 C) B9_Pink Colony D) light microscopy of B9_Pink showing their characteristic spores.**

**Figure 2:**
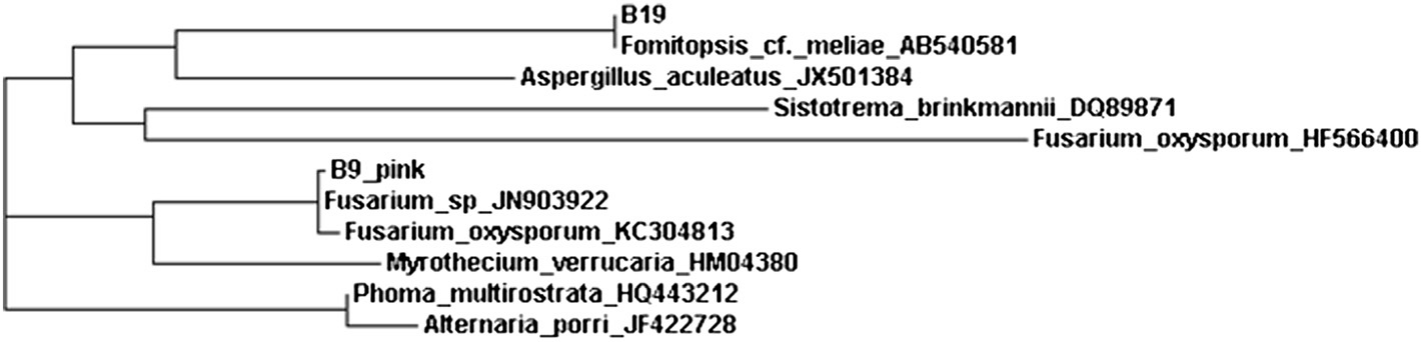
**Unrooted Phylogenetic Tree based on ITS-5.8S rDNA sequences of B9_Pink and B19 endophytic fungi associated with *Bacopa monnieri *** **showing their position with their close relatives.**

Fungi showing cytotoxic and/or antimicrobial activity ie B3, B19 and CK01 were identified on the basis of ITS-5.8S ribosomal gene sequences (Table 1). Further, ITS-5.8S ribosomal gene sequences of B3, B19 showed 99% and 100% homology with *Flavodon flavus* and *Fomitopsis cf. meliae* KYO (basidiomycota) respectively, while CK01 showed 96% homology with *Pleosporales sp.* DYXB_Y028 (ascomycota)*.* Results based on ITS-5.8S ribosomal gene sequences of B4, B16 and B9_Pink were corelated well with morphological findings (Table 1). Since B9_Pink, and B19 endophytic fungi showed most potent cytotoxic and antimicrobial activity (Figure 1), their phylogenetic position is shown in Figure 2. Contrary to the findings of Lian et al. [27], active strains in the present study were found to belong to both ascomycota and basidiomycota.

## Conclusion

In conclusion, this preliminary screening of endophytes from *Bacopa monnieri* revealed their potential to yield potent bioactive compounds that can be used in development of drugs against microbial infections and cancer. Organic extracts of B9_Pink (*Fusarium oxysporum)* and B19 (*Fomitopsis sp.*) were found to posses potent cytotoxic and antimicrobial properties, highlighting their possible potential for use in the development of anti-cancer/antimicrobial drugs, which needs to be further studied.

## Competing interests

The authors declare that they have no competing interests.

## Authors’ contributions

MK designed the study. NG extracted the fermented broth of endophytes in the supervision of PLS. SS carried out the anticancer experiments and results were analyzed by AKS. MK carried out the isolation and molecular characterization of endophytes, and drafted the manuscript. GS carried out the antimicrobial experiments. All authors read and approved the final manuscript.

## Pre-publication history

The pre-publication history for this paper can be accessed here:

http://www.biomedcentral.com/1472-6882/14/52/prepub
